# Spatio-temporal dynamic functional brain network for mild cognitive impairment analysis

**DOI:** 10.3389/fnins.2025.1597777

**Published:** 2025-06-13

**Authors:** Shipeng Wen, Jingru Wang, Wenjie Liu, Xianglian Meng, Zhuqing Jiao

**Affiliations:** ^1^Wangzheng School of Microelectronics, Changzhou University, Changzhou, China; ^2^School of Computer Information and Engineering, Changzhou Institute of Technology, Changzhou, China; ^3^School of Computer Science and Artificial Intelligence, Changzhou University, Changzhou, China

**Keywords:** dynamic functional connectivity, attention, rS-fMRI, early Alzheimer’s disease, DMNs

## Abstract

**Introduction:**

Alzheimer’s Disease (AD) is a progressive neurodegenerative disorder, with Mild Cognitive Impairment (MCI) often serving as a prodromal stage. Early detection of MCI is critical for timely intervention.

**Methods:**

Dynamic Functional Connectivity analysis reveals temporal dynamics obscured by static functional connectivity, making it valuable for analyzing and classifying psychiatric disorders. This study proposes a novel spatio-temporal approach for analyzing dynamic brain networks using resting-state fMRI. The method was evaluated on data from 85 subjects (33 healthy controls, 29 Early Mild Cognitive Impairment (EMCI), 23 AD) from the ADNI dataset.

**Results:**

Our model outperformed existing techniques, achieving 83.9% accuracy and 83.1% AUC in distinguishing AD from healthy controls.

**Discussion:**

In addition to improved classification performance, key affected regions such as left hippocampus, the right amygdala, the left inferior parietal lobe, the left olfactory cortex, the right precuneus, and the insula, were identified-areas known to be associated with memory function and early Alzheimer’s pathology. These findings suggest that dynamic connectivity analysis holds promise for non-invasive and interpretable early-stage diagnosis of AD.

## 1 Introduction

Brain networks and functional connectivity are fundamental to studying brain disorders, enabling the exploration of complex relationships between brain dysfunction and behavioral phenotypes (Desikan et al., 2006). Alzheimer’s disease (AD) is the most prevalent progressive neurodegenerative disorder, accounting for 50%–80% of dementia cases (Glover, 2011; Gonzalez-Castillo et al., 2021). Despite extensive research, there is currently no effective treatment, and the disease leads to a marked decline in quality of life. After the age of 65, its incidence doubles approximately every 5 years, and it is projected that by 2050, one in 85 individuals will be affected. Mild cognitive impairment (MCI), a precursor to AD, has an annual conversion rate of 10%–15%, with over 50% progressing to AD within 5 years. Given the high conversion rate and increasing lifespan, reducing MCI-to-AD progression through pharmacological and non-pharmacological interventions has become a research focus, making early and accurate MCI identification crucial (Kim et al., 2010; Lam et al., 2013; Loewenstein et al., 2006; Richards and Berninger, 2008; Wise and Preston, 2010).

Functional magnetic resonance imaging (fMRI) is a non-invasive technique that measures blood-oxygen-level-dependent (BOLD) signals, providing insight into neural activity across different brain regions. It has been widely used to investigate functional connectivity abnormalities in patients with MCI, a transitional stage between normal aging and Alzheimer’s disease. However, despite utility, significant challenges remain in accurately constructing dynamic brain networks and capturing subtle spatiotemporal functional abnormalities from fMRI data (Richards and Berninger, 2008). Dynamic functional connectivity (dFC) analysis reveals temporal dynamics obscured by static functional connectivity (sFC), making it valuable for analyzing and classifying psychiatric disorders. Studies have highlighted the potential of dFC in uncovering temporal dynamics and abnormal connectivity patterns, though limitations such as small sample sizes and lack of longitudinal analysis persist (Bahrami et al., 2022; Li et al., 2020; Li et al., 2021; Wang Z. et al., 2022; Yang et al., 2022). Huang et al. (2022) conducted a systematic review summarizing the use of dFC in schizophrenia research. They emphasized its potential for revealing temporal brain dynamics and detecting abnormal connectivity patterns. They also highlighted the limitations of current studies and proposed future directions, including increasing sample sizes and employing more complex analytical methods. However, their study focused only on cross-sectional research and did not address the temporal evolution of dFC during disease progression, such as the transition of connectivity patterns from early to chronic stages. Chen et al. (2025) applied graph neural networks (GNNs) to analyze dFC in patients with schizophrenia, constructing dynamic brain networks by sliding window technique and extracting features with GNN models. Their results demonstrated that GNN models outperformed traditional machine learning methods in classification accuracy, showcasing the potential of GNNs in dFC analysis of schizophrenia. Nevertheless, their study used only the mean of sliding windows to construct dFC, failing to capture higher-order dynamic characteristics. Chen et al. (2024) employed a sliding window technique to analyze dFC in schizophrenia patients, segmenting resting-state fMRI (rs-fMRI) data into multiple time windows and calculating dFC for each segment. Their findings revealed significant differences in dFC patterns between patients and healthy controls, indicating that dFC analysis can provide valuable insights into the neurobiological mechanisms of schizophrenia. This further confirmed the importance and potential of dFC analysis in mental disorder research. However, their study only reported group-level differences in dFC, which may lead to weak clinical associations.

Graph neural networks have shown promise in MCI classification by automatically learning and integrating features from adjacent nodes, outperforming traditional machine learning methods (Wu et al., 2020a). GNNs extend deep learning to non-Euclidean domains, enhancing feature extraction and aggregation in graph-structured data. Recent studies have applied GNNs to multi-modal MRI and EEG data, capturing complex brain network relationships and improving MCI classification accuracy. However, challenges such as noise interference, static connectivity analysis, and high computational complexity remain (Song et al., 2023; Veličković et al., 2017). Zhang Y. et al. (2023) constructed a graph structure from multimodal MRI data and analyzed it using GNNs, precisely capturing the complex relationships within brain networks and successfully identifying topological abnormalities in the functional networks of MCI patients. However, their study did not perform cross-modal feature selection, leading to noise interference in classification performance due to high-dimensional inputs. Demir et al. (2021) focused on Electroencephalogram (EEG) data, using GNNs to build graph structures and explore functional connections between different brain regions, providing strong support for the precise identification of MCI. Nevertheless, their study only constructed static functional connectivity graphs without leveraging the high temporal resolution of EEG to capture dynamic interactions. Li et al. (2024) proposed an innovative GNN model based on multimodal data fusion, integrating structural MRI, functional MRI, and EEG data to comprehensively capture structural and functional information of the brain, thereby further improving the accuracy of MCI classification. However, their use of decision-level fusion may result in the loss of early interaction information between modalities, such as structure-function coupling. An et al. (2020) utilized GNNs to analyze dFC data in MCI patients, constructing dynamic brain networks to precisely capture the temporal dynamics of brain activity, offering a new perspective for MCI classification. However, their study only used the mean of sliding windows and failed to capture non-linear temporal patterns of dynamic connectivity. Zhang D. et al. (2023) applied GNNs to extract key features from fMRI, constructing graph structures of brain networks to further reveal functional connections between brain regions, thereby validating the effectiveness and superiority of GNNs in MCI classification. However, their study only analyzed static functional connectivity without utilizing the temporal dimension of fMRI.

Gao and Ji (2019) proposed a Graph U-Net model that employs graph pooling techniques to hierarchically cluster nodes within brain networks, effectively extracting features at different levels. This method demonstrated superior performance in brain network analysis and the ability to identify abnormal patterns, providing new tools and approaches for brain network research. However, despite its excellent feature extraction capabilities, the predefined pooling layers of the Graph U-Net model cannot adapt to the modular structures of different brain networks, such as disease-specific community divisions. Hu et al. (2023) introduced a self-attention-based graph pooling method that achieved high accuracy in identifying brain network abnormalities. This approach also better captured topological features, offering a new perspective for brain network analysis. Nevertheless, the study effectively extracted key features, the use of multi-layer attention mechanisms may increase computational complexity. Additionally, attention aggregation could homogenize node features, potentially reducing local specificity. Wu et al. (2024) applied graph pooling techniques to analyze dynamic brain networks, constructing dynamic brain networks using a sliding window technique and extracting key nodes and features with graph pooling methods. This approach effectively captured the temporal dynamics of brain activity, providing a new perspective for dynamic brain network analysis. However, their study only used the mean of sliding windows to represent dynamics without modeling state transitions (e.g., Markov chains) or temporal dependencies, thereby affecting a comprehensive understanding of dynamic brain networks.

This study proposes a Dynamic Graph Recurrent Neural Network (Dynamic-GRNN) model for brain network analysis, combining sliding windows and Slide Piecewise Aggregation (SPA) with Pearson Correlation Coefficient (PCC) to construct dynamic brain networks. The model employs spatiotemporal encoding to capture dynamic interactions and introduces self-attention graph pooling (SAGPooling) to select key nodes, addressing issues like noise sensitivity and static connectivity limitations. Evaluated on the ADNI dataset, the model achieved an 83.9% accuracy in subjects with cognition normal (CN)/AD classification, providing a high-precision, interpretable method for early neurodegenerative disease diagnosis (Gadgil et al., 2020; Liu et al., 2023; Smith et al., 2012).

The main contributions of this paper can be summarized as follows:

1) SPA-PCC Joint Modeling: Combining SPA with sliding windows to enhance node features, suppress noise, and improve temporal expression.
2) Dynamic-GRNN Spatiotemporal Encoding: Jointly modeling brain network functionality and time series dynamics.
3) Temporal SAGPooling: Dynamically selecting Top-K nodes based on cross-temporal attention weights to identify persistently abnormal brain regions, improving classification accuracy.

## 2 Materials and methods

### 2.1 Core nodes with multiple feature combinations

In recent years, a large number of researchers have studied the core nodes of complex networks. Many literatures have also systematically summarized the related research results and given three main evaluation indexes for identifying core nodes: including degree centrality, betweenness centrality and closeness centrality.

The calculation of degree centrality is based on the degree itself, the degree ki of a node indicates the number of neighboring edges of the node *v*_*i*_, and a larger value indicates a larger degree, and a larger degree indicates that the node undertakes more information transfer and conversion work in the network. Therefore, it is one of the common indicators for evaluating the importance of a node and the calculation formula is shown in Eq. (1).


(1) $$K_{i}=\sum_{j=1,j\neq i}^{N}K_{i\,j}$$

Where *N* is the total number of nodes and *K*_*ij*_ denotes the number of connected edges between node *i* and *j*.

Median centrality defines the degree of centrality of a node in terms of information flow. The median centrality of a node *v*_*i*_ is the ratio of the number of shortest paths passing through the node to the total number of shortest paths of the node pair. It reveals the importance of the brain area node in the whole process of information flow transmission in the network. From the view of the global characteristics of the network, it laterally reflects the global control ability of this node. The larger the node median center value, the more likely the node is to be a bridge between other nodes. Thus it is another common metric for evaluating the importance of a node, which is calculated by the following formula is shown in Eq. (2).


(2) $$b_{i}=\frac{1}{\left(N-1\right)\,\left(N-2\right)}\,\sum_{\begin{array}{c} h,j\in N\\ h\neq j,h\neq i,j\neq i \end{array}}\frac{r_{h\,j}^{\left(i\right)}}{r_{h\,j}}$$

where *N* is the total number of nodes, *r*_*hj*_ denotes the number of shortest paths between node *h* and *j*, and rh⁢j(i) is the number of paths that pass through node *i* among all shortest paths between node *h* and *j*.

The calculation of closeness centrality is based on the concept of the shortest path, which is the reciprocal of the sum of the shortest paths from the node to all other nodes. It focuses on expressing the degree of difficulty of a node to other nodes. In contrast to the local nature of degree centrality, proximity centrality reflects the global structure of the network. The larger its value, the closer the node is to other nodes, and its calculation formula is as follows:


(3) $$C_{i}=\frac{1}{\sum_{j=1,j\neq i}^{N}D_{i\,j}}$$

where *N* is the total number of nodes in the network and *D*_*ij*_ is the shortest path distance between nodes *i* and *j*.

Degree centrality is the most commonly used method for identifying key nodes. Median centrality is capable of evaluating the role of nodes in the information transfer process of brain networks. Although the application frequency of proximity centrality is lower than that of degree centrality and median centrality, it is able to evaluate the contribution of individual nodes to the whole network from the perspective of network efficiency. In this study, a composite equation is constructed through degree centrality, median centrality and proximity centrality, as shown in Eq. (4).


(4) $$C\,o\,u\,n\,t=\sum_{i=1}^{m}t_{i}\,p_{i}$$

Where, *m* is the number of composite parameters selected, *t*_*i*_ is the weight of the parameters and *p*_*i*_ is the value of the graph theory parameters.

### 2.2 Dynamic functional brain network study

The sliding window technique is a classical time series feature extraction method, which is widely used in brain network construction to solve practical problems in various fields (Wein et al., 2022). In this study, the sliding window technique is used to construct node and edge features to fully utilize the temporal information in fMRI. Assuming that there is a time series of length *K*. In order to extract the temporal features, it is necessary to choose a reasonable window width *W* and a sliding step size *s*. By moving the sliding window over the time series *K* with a given sliding step size *s*, the whole time series is divided into *m* time slices. Each time slice starts at *t* and ends at *t*+*W*. It should be emphasized that *t* can only be an integer. The total number of time slices *m* can be calculated as follows:


(5) $$m=\frac{K-w}{s}+1$$

To better process fMRI, this study extends the segmented aggregation approximation method and proposes the SPA method. The method applies continuous time series to discrete time series of fMRI to extract node features, which provide effective inputs for subsequent GNN analysis. Specifically, for the fMRI signals of each brain region, the signal values within each time slice are averaged as shown in Eq. (6).


(6) $${\bar{B\,O\,L\,D}}^{n}=\frac{1}{w}\,\sum_{i=1}^{w}B\,O\,L\,D_{i}^{n}$$

where *BOLD* denotes the signal value during the nth time, ${\bar{B\,O\,L\,D}}^{n}$ denotes the average signal value during the nth time slice, and W denotes the width of the time slice which is the number of signal points contained in each time slice.

Through the above averaging process, *BOLD* are generated for each brain region. Each signal corresponds to a time slice. Concatenating these *BOLD* signals in the order of time to form a node feature vector. The feature vectors not only simplify the data, but also retain the dynamic change information of the time series, which helps to capture the dynamic properties of the network and provides an effective input for the subsequent GNN analysis.

In brain networks, nodes denote brain regions or regions of interests (ROIs), and edges denote functional connectivity between nodes. After SPA approach which divides the time series into multiple time slices, calculate the correlation between nodes from the data within each time slice. For nodes *v_i_* and *v_j_*, the PCC is utilized to calculate their edge feature vectors in the current time period as shown in Eq. (7).


(7) $$r_{v_{i}\,v_{j}}\,\left[k\right]=$$

$$\frac{\sum_{g=1}^{w}\left(B\,O\,L\,D_{g}^{v_{i}}-{\bar{B\,O\,L\,D}}^{v_{i}}\right)\,\left(B\,O\,L\,D_{g}^{v_{j}}-{\bar{B\,O\,L\,D}}^{v_{j}}\right)}{\sqrt{\sum_{g=1}^{w}{\left(B\,O\,L\,D_{g}^{v_{i}}-{\bar{B\,O\,L\,D}}^{v_{i}}\right)}^{2}}\,\sqrt{\sum_{g=1}^{w}{\left(B\,O\,L\,D_{g}^{v_{j}}-{\bar{B\,O\,L\,D}}^{v_{j}}\right)}^{2}}}$$

where $B\,O\,L\,D_{g}^{v_{i}}$ and $B\,O\,L\,D_{g}^{v_{i}}$ denote the signal values of node *v_i_* and node *v_j_*, respectively, at time point *g*. ${\bar{B\,O\,L\,D}}^{v_{i}}$ and ${\bar{B\,O\,L\,D}}^{v_{j}}$denote the average signal values of *v_i_* and *v_i_*, respectively, within the current time slice. *w* denotes the width of the time slice.

For the generated PCC values of *m* time slices, the PCC values are scaled to the range of 0–1 using Min-Max normalization. The normalization formula is shown in Eq. (8). After that, these PCC values are arranged into edge feature vectors *L* as shown in Eq. (9).


(8) $$r_{v_{i}\,v_{j}}\,\left[k\right]=\frac{r_{v_{i}\,v_{j}}\,\left[k\right]-\min\left(r_{v_{i}\,v_{j}}\right)}{\max\left(r_{v_{i}\,v_{j}}\right)-\min\left(r_{v_{i}\,v_{j}}\right)}$$

(9) $$L\,\left(v_{i},v_{j}\right)={\left\{r_{v_{i}\,v_{j}}\,\left[1\right],r_{v_{i}\,v_{j}}\,\left[2\right],\ldots ,r_{v_{i}\,v_{j}}\,\left[m\right]\right\}}^{T}\in R^{m}$$

The PCC provides a metric to quantify the strength of connections between nodes, which helps in subsequent network analysis and machine learning tasks. While the normalization process makes the PCC value between 0 and 1, which facilitates comparisons between different time slices and subsequent processing (Gadgil et al., 2020; Hu et al., 2024).

### 2.3 Overall analysis framework

**FIGURE 1:**
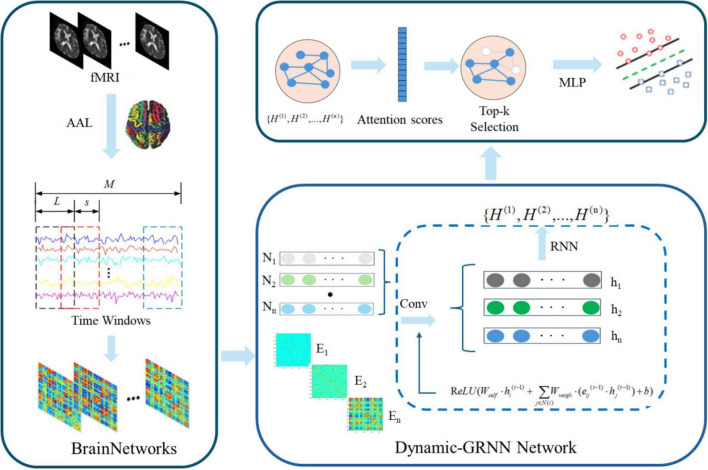
Dynamic Graph Recurrent Neural Network (Dynamic-GRNN) framework for dynamic brain network analysis.

Figure 1 illustrates the proposed framework, which is summarized into: (1) Introduce sliding window algorithm to divide fMRI into multiple overlapping time segments (Zheng et al., 2023). Calculating the node features of brain regions within each time segment using the SPA method, and calculating the edge features between brain regions using the PCC method to construct a dynamic functional brain network; (2) Introduce the Dynamic-GRNN network to deal with time series issues in graph-structured data, capturing dynamic changes in time series while maintaining temporal synchronization (Seo et al., 2018); (3) Introduce the SAGPooling method, which selects the Top-K most important nodes across the whole brain based on temporal attention weights, more flexibly capturing dynamic key brain regions across hemispheres, and reassembling the pooled nodes to be input into subsequent classifiers (Gu et al., 2020; Zhu et al., 2022).

#### 2.3.1 Dataset and preprocessing

Table 1 shows the research from public data sets of Alzheimer’s disease Neuroimaging plan (Alzheimer’s diseases, Neuroimaging Initiative, ADNI)^1^ collected 85 cases of the participants. All of them had multimodal data (fMRI and DTI), including 33 HC subjects, 29 EMCI subjects, and 23 AD subjects. Group classification was based on ADNI diagnostic labels. Cognitive status was primarily assessed using the Mini-Mental State Examination (MMSE), a 30-point questionnaire widely used to evaluate global cognitive function, including orientation, attention, memory, language, and visuospatial abilities. EMCI participants had subjective memory complaints and mild objective memory impairment, with preserved general cognitive function (MMSE ≥ 24). AD participants exhibited more severe memory and cognitive decline (MMSE typically ≤ 26). Inclusion criteria required the availability of both fMRI and DTI data and a confirmed ADNI diagnosis of HC, EMCI, or AD. Exclusion criteria included major neurological disorders (e.g., stroke, Parkinson’s disease), psychiatric conditions (e.g., major depression, schizophrenia), recent substance abuse, significant head trauma, or unstable medical conditions that could impact brain function or structure.

**TABLE 1:** Basic information on collected data.

Subject	HC	EMCI	AD	*P*
Number	33	29	23	–
Gender (M/F)	12/21	14/15	9/14	< 0.001
Age (Mean ± sd)	73.88 ± 7.15	74.52 ± 7.30	74.34 ± 8.14	< 0.001
APOE4 (0/1/2)	19/13/1	20/9/0	13/7/3	< 0.001
MMSE (mean ± sd)	29.15 ± 1.13	28.52 ± 1.45	21.78 ± 1.89	< 0.001
Edu (mean ± sd)	16.55 ± 2.34	16.31 ± 2.56	14.96 ± 1.90	< 0.001

M, male; F, female; 0/1/2, number of APOE ε4 alleles; Mean ± sd, mean and standard deviation.

Quality control was performed on the data of these subjects, and experiments were conducted using the data after quality control. All neuroimaging data were obtained using the SIEMENS 3T MRI scanner. After *T*-test, Gender, Age, Apolipoprotein E4 (APOE4), MMSE and Education level (Edu) among the three different levels of MCI met *p* < 0.001, indicating that the differences were statistically significant.

Each participant contributed 198 imaging scans. Ultimately, a total of 16,830 scans were allocated into training, validation, and test sets following a 6:2:2 ratio.

Functional magnetic resonance imaging data were preprocessed by SPM12 (Statistical Parametric Mapping, a widely used neuroimaging software for spatial normalization and statistical analysis) (Penny et al., 2007) and DPARSF (Data Processing Assistant for Resting-State fMRI, a user-friendly toolbox that integrates SPM functions with specific preprocessing pipelines for resting-state analysis) (Chao-Gan and Yu-Feng, 2010) tools.

The key processes were the following steps: (1) The image data in the original DICOM format were converted to NIFTI format; (2) The images of the first 10 time points of each subject were manually removed; (3) The scanning time of all the slices was corrected for slice time consistency; (4) The images with head movement of more than 2.5 mm or rotation of more than 2.5 degrees were removed to correct for head movement during scanning; (5) White matter signals and cerebrospinal fluid signals of head movement were set as the main noise covariates to reduce the effect of noise on the scanning results and to minimize the effect of biological artifacts at the same time; (6) Brain images with different morphologies were aligned to a standard template and matched with T1-weighted images; (7) A Gaussian kernel of 4 × 4 × 4 mm was applied to the images for spatial smoothing; (8) The linear trend of the data was removed, and filters ranging from 0.01 to 0.1 Hz were applied to eliminate the interference of low and high frequency noise. The brain was divided into 90 functional areas using an Anatomical Automatic Labeling template. The BOLD signal time series and Pearson correlation matrix were extracted from these regions.

#### 2.3.2 Updating node states

After getting the nodes and their features by the slice window method, the node matrix *N* obtained by processing using the SPA method, and the edge features obtained by the PCC method. This study obtains the input data of the GRNN which called the node features and the dynamic edge features. Specifically, the feature vectors of each node in each time window form the node feature matrix **X** ∈ *R^n × m × d^*, where *n* is the number of nodes, m is the number of time windows, and d is the feature dimension in each time window. The feature vectors of each edge in each time window form the edge feature matrix **E** ∈ *R^e× m × f^*, where *e* is the number of edges and *f* is the feature dimension in each time window. Then the node and edge features are aggregated and updated in chronological order, which is explained as follows: at each time step *t*, the state$h_{i}^{\left(t\right)}$ of node *i* is jointly determined by its state $h_{i}^{\left(t-1\right)}$ at the previous time step *t*–1 and the state $h_{j}^{\left(t-1\right)}$ of its neighbor nodes, and the update equation as follows:


(10) hi=(t)σ(W⋅(hi+(t-1)∑j∈N⁢(i)Ei⁢j⋅(t-1)hj)(t-1)+b)

where $h_{i}^{\left(t\right)}$ is the state of node i at time t, $h_{i}^{\left(t-1\right)}$ is the state of node i at time step t–1, $h_{j}^{\left(t-1\right)}$ is the state of node j at time step t–1, j is the neighbor of node i, $E_{\mathrm{ij}}^{\left(t-1\right)}$ is the feature of the edges between node i and j at time step t–1. W is the learned weight parameter, b is the bias vector, and σ is the ReLU activation function.

By multiplying $h_{i}^{\left(t-1\right)}$ with the weight matrix ***W***,$h_{i}^{\left(t-1\right)}$could be adjusted by the effect of the node’s own state on the current state. The state of node *j*, which is a neighbor of node *i* at the previous time step *t*−1, denoted as $h_{j}^{\left(t-1\right)}$, is also an important part of the node state update. It reflects the dynamic information of the neighboring nodes of node *i* at the previous time step. The $E_{i\,j}^{t}$reflects the strength of the relationship between node i and j which is also used to adjust the neighbor node states. By multiplying $h_{j}^{\left(t-1\right)}$ with the edge feature $E_{i\,j}^{t-1}$, the influence of neighbor node states on the current state could be adjusted. By multiplying the states of all neighboring nodes $h_{j}^{\left(t-1\right)}$ with their respective edge features $E_{i\,j}^{\left(t-1\right)}$ and then summing them up, the state information of the neighboring nodes can be aggregated. This aggregation process captures the dynamic changes of node *i* and its neighboring nodes from the previous time step, further knows the impact of these changes on node *i*. For example, if $E_{i\,j}^{\left(t-1\right)}$ is heavier, it means that the relationship between node *i* and *j* is stronger, the state of neighbor node *j* has a greater impact on node *i*. While maintaining time-synchronous relationships, GRNN utilizes node features and edge features to capture dynamic changes in the time series through recursive joins and node state updating. By performing recursive connections and node state updates in all time windows, GRNN eventually synthesizes a complete graph structure. This graph structure contains node states and edge features for all time windows and is capable of capturing dynamic changes in the entire time series (Yu et al., 2018). The final graph structure is used for subsequent classification tasks (Wu et al., 2020b).

#### 2.3.3 SAGPooling layer

The high dimensionality of node features in the original graph leads to increased difficulty in extracting global information, whereas introducing a node pooling layer between graph convolution layers to obtain subgraphs with fewer nodes and features can improve the generalization ability of the model. Recent research (Lee et al., 2019; Zhang et al., 2020) results have shown that some brain regions are more important than others in predicting brain diseases, and the use of node pooling layers to reduce the size of the graph and retain only some important nodes is crucial. In addition, the pooling layer reduces the size of the network parameters. Thus, coarsening the node representation on the graph provides a better graph-level representation. We propose a topology-based attention pooling module, which adaptively learns the importance of nodes. The Top-K mechanism is used to select a group of nodes dynamically for pooling, which not only considers the node characteristics, but also preserves the graph topology. In such a way that node characteristics are taken into account and the graph topology is preserved.

The representation of the introduced attention pooling operation can be described as Eq. (11). For a given node feature *X*^(2)^ ∈ *R^n×c^*, use a GraphConv layer with only one output channel to calculate the attention score *z* ∈ *R^n ×1^* that encodes the graph topology information. Attention scores are then processed using the tanh function to obtain the final attention weights. A fraction of the nodes of the regional brain graph are retained using the Top-K node selection strategy, where *K* is the proportion of nodes in the new graph. Among the selected *K* nodes, the idx values of the *K* largest taken values of the output ordering of the nodes are obtained based on the calculation result *z*. Next, element-wise multiplication of the indexed features *X*^(2)^ and z are multiplied element-by-element to capture the pool node features ${\hat{X}}^{\left(2\right)}$. Finally, a row-by-element multiplication of *A*(*idx*,*idx*) row and column extraction is performed to form a new adjacency matrix $\tilde{A}$. In summary, through the graph pooling layer, the input graph (*V*_(*k*)_,*E*_(*k*)_) is updated, and the output graph is (*V*_(*k* + 1)_,*E*_(*k* + 1)_). Attention to the graph pooling layer reduces the number of parameters and selects the nodes that are important for classification, which is crucial for discovering brain regions associated with diseases, while making the classification results more interpretable.


(11) $$\begin{array}{c} z=G\,r\,a\,p\,h\,C\,o\,n\,v\,\left(X^{\left(2\right)},A\right),\\ a=\text{softmax}\,\left(z\right)\\ \tilde{z}=\tanh\left(a\right),\\ \mathrm{idx}=t\,o\,p\,k\,\left(\tilde{z},k\right),\\ {\hat{X}}^{\left(2\right)}=X^{\left(2\right)}\,\left(\mathrm{idx},:\right),\\ {\tilde{X}}^{\left(2\right)}={\hat{X}}^{\left(2\right)}⊙\tilde{z},\\ \tilde{A}=A\,\left(\mathrm{idx},\mathrm{idx}\right). \end{array}$$

## 3 Results

### 3.1 Experimental implementation and setting

The experiments in this study were performed on a computer equipped with a single RXT4080super GPU. Model construction and algorithm training and testing were conducted using the PyTorch deep learning framework. The labels of all subjects were shuffled, and parameters were determined via the optuna algorithm. The experimental design employed a leave-one-out cross-validation approach, with 10 iterations of leave-one-out averaging and 100 iterations per round. The batch size was set to 32. All models were optimized using the Adam optimizer with a learning rate of 0.001. For the network models, ReLU was selected as the activation function in the two-layer perceptron. Dropout regularization was applied to the graph pooling layer to prevent overfitting, with nodes randomly dropped at an optimal probability during training. An early stopping strategy was implemented to avoid overfitting during training, with a patience level of 50. Specifically, the early stopping was triggered when the loss value remained within a set threshold for 50 consecutive counts (Prechelt, 2002).

### 3.2 Evaluation metrics

To thoroughly investigate the core issues addressed in this study, when dealing with labeled data, category 1 and category 0 in the binary classification task were defined as the positive class and negative class. In evaluating model performance using the test dataset, the following four key classification scenarios were considered: True Positive (TP): The number of samples that actually belong to the positive class and are correctly predicted by the model as positive; False Negative (FN): The number of samples that actually belong to the positive class but are incorrectly predicted by the model as negative; False Positive (FP): The number of samples that actually belong to the negative class but are incorrectly predicted by the model as positive; True Negative (TN): The number of samples that actually belong to the negative class and are correctly predicted by the model as negative.

These four classification scenarios were carefully examined to comprehensively assess the model’s classification effectiveness. The counts of true positives and true negatives directly reflect the model’s accuracy, while the counts of false positives and false negatives reveal the model’s misclassifications. The introduction of these metrics provides a solid foundation for the quantitative evaluation of model performance, enhancing the objectivity and reliability of the results. In subsequent tests and analyses, the proposed model in this study demonstrated superior performance in handling labeled data based on these evaluation criteria. Accuracy, a commonly used evaluation metric for classification problems, reflects the ratio of correctly classified samples to the total number of samples. For traditional balanced classification problems, accuracy is a good measure of classification algorithm performance. It is calculated as the ratio of correctly classified data points to the total number of data points, with values ranging from 0 to 1. The closer the value is to 1, the better the model performs. The calculation formula is given in Eq. (12).


(12) $$A\,c\,c\,u\,r\,a\,c\,y=\frac{T\,P+T\,N}{T\,P+F\,P+F\,N+T\,N}$$

Sensitivity indicates the proportion of all people with the disease who are correctly classified by the model, the higher the sensitivity the lower the probability of missing the diagnosis; Precision is the proportion of samples predicted to be positive by the model that are actually positive; Recall is the proportion of all actual positive samples that are correctly predicted to be positive by the model; F1-score denotes the precision and Recall; AUC denotes the area under the ROC curve, which provides a numerical assessment of the overall performance of the model. In this study, ACC was mainly used as a reference index.

### 3.3 Network topology attribute validation

To address the differences in node importance values described by the three single-feature core node identification methods, a threshold was established for each method. A node was considered a core node under a specific evaluation metric if its importance value exceeded the sum of the mean and variance of the importance values of all nodes for that method. For instance, under the degree centrality metric, a core node was defined as one with a degree value greater than the sum of the mean degree value and the variance of degree values across all nodes. Based on this logic, the results of the experiment and the number of core nodes selected are shown in Table 2.

**TABLE 2:** Number of core nodes selected under different graph theoretic approaches.

Methods	Mean	Variance	Threshold	Number of nodes
Degree centrality	14.22	6.36	20.58	13
Betweenness centrality	104.73	99.34	204.27	10
Closeness centrality	0.53	0.06	0.59	13

**FIGURE 2:**
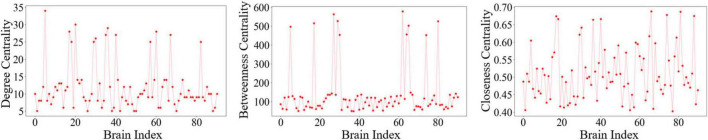
Changes in core node metrics for three single features: **(a)** Degree centrality. **(b)** Betweenness centrality. **(c)** Closeness centrality.

The variation curves of these three single-feature core node metrics are illustrated in Figure 2.

The constructed composite equation is expressed as follows:


(13) $$C\,o\,u\,n\,t=k_{1}\,P_{1}+k_{2}\,P_{2}+k_{3}\,P_{3}$$

**FIGURE 3:**
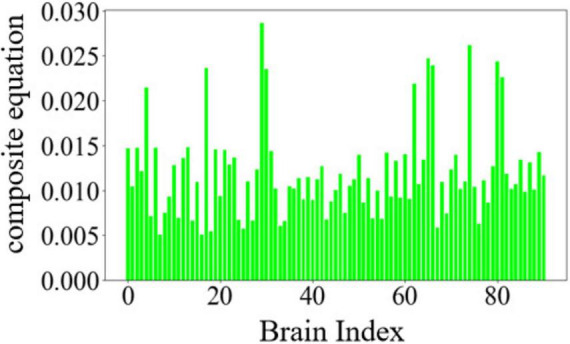
Importance of nodes in composite equations.

Where *P*_1_ represents the normalized value of degree centrality, *P*_2_ represents the normalized value of betweenness centrality, and *P*_3_ represents the normalized value of closeness centrality. *k*_1_, *k*_2_, *k*_3_ are the absolute values of the mean slopes of the degree centrality curve, betweenness centrality curve, and closeness centrality curve, respectively. The importance of nodes was re-ranked using the above composite equation. The variation of node importance based on the composite equation is illustrated in Figure 3.

### 3.4 Model comparison

This study proposes a Dynamic-GRNN model based on dynamic dFC and attention pooling layer for the early identification of MCI. Its classification performance was systematically evaluated on the ADNI dataset. The results of the comparative experiments were divided into three groups, and the respective experimental results are shown in Tables 3–5. The results demonstrate that the model outperforms existing methods in several key metrics, thereby validating the effectiveness of dynamic brain network analysis and spatiotemporal attention modeling. Specifically, under the same experimental conditions, the classification accuracy (ACC) of this paper’s model reaches 83.9%, which is a 2.0% improvement over the suboptimal model Temporal-BCGCN (81.9%). It is improved by 4.1% and 7.5% over traditional support vector machine (SVM, 79.8%) and brain graph neural network (BrainGNN, 76.4%), respectively. This result highlights the superiority of dFC over sFC in capturing changing temporal signals, suggesting that temporal dynamic features play a key role in capturing early pathological patterns of neurodegenerative diseases (Zhu et al., 2024).

**TABLE 3:** Compare with state-of-the art methods on Alzheimer’s disease (AD)/CN subjects.

Classifiers	Condition	ACC	Recall	PREC	F1-score	AUC
SVM	sFC	0.798	0.745	0.760	0.747	0.752
BrainGNN	sFC	0.764	0.679	0.700	0.679	0.736
GCN	dFC	0.764	0.713	0.721	0.711	0.745
CRNN	dFC	0.785	0.739	0.756	0.744	0.760
Temporal-BCGCN	dFC	0.819	0.734	0.795	0.762	0.811
Proposed method	dFC	0.839	0.816	0.805	0.810	0.831

**TABLE 4:** Compare with state-of-the art methods on mild cognitive impairment (MCI)/CN subjects.

Classifiers	Condition	ACC	Recall	PREC	F1-score	AUC
SVM	sFC	0.723	0.684	0.698	0.691	0.715
BrainGNN	sFC	0.692	0.637	0.663	0.649	0.681
GCN	dFC	0.708	0.661	0.679	0.670	0.703
CRNN	dFC	0.733	0.695	0.708	0.701	0.729
Temporal-BCGCN	dFC	0.768	0.712	0.737	0.724	0.762
Proposed method	dFC	0.785	0.753	0.762	0.757	0.792

**TABLE 5:** Compare with state-of-the art methods on Alzheimer’s disease (AD)/mild cognitive impairment (MCI) subjects.

Classifiers	Condition	ACC	Recall	PREC	F1-score	AUC
SVM	sFC	0.683	0.625	0.647	0.636	0.672
BrainGNN	sFC	0.652	0.593	0.611	0.602	0.638
GCN	dFC	0.667	0.614	0.628	0.621	0.658
CRNN	dFC	0.697	0.653	0.672	0.662	0.693
Temporal-BCGCN	dFC	0.732	0.671	0.703	0.686	0.741
Proposed method	dFC	0.753	0.714	0.728	0.721	0.769

Further analysis of the model’s robustness to class imbalance revealed a recall rate of 81.6% and an F1-score of 81.0%, both of which significantly outperformed comparison methods. For instance, Temporal-BCGCN achieved a recall rate of 73.4% and an F1-score of 76.2%. These results demonstrate the model’s enhanced ability to accurately identify the small number of MCI patients in the dataset. Additionally, the leading performance in AUC metrics indicates that the model excels in distinguishing the decision boundary between MCI and healthy individuals, thus offering greater clinical applicability.

### 3.5 Ablation study

Systematic ablation experiments were conducted in this study to verify the necessity of the dFC, GRNN, and SAGPooling modules, as well as their synergistic effects in the AD/CN classification task. The experimental design included three sets of comparison models: (1) GRNN with input sFC; (2) dFC combined with GRNN without pooling; and (3) the complete model. As shown in the Table 6, on the ADNI dataset, the complete model achieved an ACC of 83.9%, representing an 8.7% improvement over the 75.2% of the GRNN-only model and a 3.8% improvement over the 80.1% of the dFC-GRNN model without pooling. These results demonstrate the incremental contributions of each module.

**TABLE 6:** Performance metrics for experiments using individual components.

Model	dFC	GRNN	SAGPooling	AD/CN
GRNN	–	√	–	0.752
dFC + GRNN	√	√	–	0.801
Proposed	√	√	√	0.839

Compared to sFC, dFC captured the temporal dynamics of the brain network through a sliding window, significantly enhancing the model’s sensitivity to AD pathological features. The ACC increased from 75.2% to 80.1% after incorporating dFC. GRNN achieved deep integration of spatiotemporal features through the joint design of spatial encoding and temporal recursive layers. Experiments showed that the ACC fluctuation range of GRNN was significantly smaller than that of non-temporal models, verifying its ability to suppress transient noise. Additionally, GRNN’s capacity to model long-range dependencies, such as the progressive degradation of hippocampus-prefrontal connections, was enhanced under dFC conditions, as evidenced by an increase in Recall. This indicates that temporal modeling is crucial for identifying the minority class samples, i.e., AD patients.

After introducing SAGPooling, the model filtered key nodes across hemispheres (posterior cingulate gyrus, medial temporal lobe) based on global importance scores. The ACC further improved to 83.9% while reducing computational effort, highlighting the module’s ability to focus on pathologically relevant regions. Statistical validation of the ablation experiments further confirmed the significance and effect size of the performance improvements attributed to each module.

## 4 Discussion

### 4.1 Discussion on improved GRNNs

Traditional Graph Recurrent Neural Networks (GRNNs) commonly use sFC as the foundational framework for brain network modeling. For instance, the approach proposed by Lee et al. (2024) calculates the PCC using the average BOLD signal over the entire time period to construct a fixed adjacency matrix. A GCN is then employed to extract spatial topological features, followed by feeding node time series into an LSTM for classification. While this method achieves a baseline accuracy (ACC = 97.3%) in AD classification tasks, it reveals inherent limitations of static models: sFC cannot capture the temporal dynamics of functional connectivity between brain regions, leading to decoupled optimization of spatial feature extraction and time series modeling, thereby ignoring the time-varying patterns of node features.

The proposed Dynamic Graph Recurrent Neural Network (dynamic-GRNN) overcomes these limitations through a dual optimization mechanism. In the spatial dimension, it introduces dynamic PCC connection strength as an adaptive weight regulator [in Eq. (14)], enabling real-time updates of neighbor node information propagation weights:


(14) hi=(t)σ(Ws⁢e⁢l⁢f⋅hi+(t-1)∑j∈N⁢(i)Wn⁢e⁢i⁢g⁢h(ei⁢j⋅(t-1)hj)(t-1)+b)

In the temporal dimension, a hidden state recurrence equation hi=(t)f(hi,(t)xi)(t) is constructed to model the slow evolution of brain networks through historical state dependencies. This spatiotemporal joint optimization mechanism effectively addresses the decoupling of spatial and temporal processing in traditional GRNNs. Simulation experiments demonstrate that dynamic-GRNN improves feature representation capability on fMRI data by 8.7%, validating the effectiveness of the dynamic modeling mechanism.

### 4.2 Discussion of GRNN in proposed framework

Within the overall analysis framework, dynamic-GRNN serves as the core component for deep spatiotemporal feature integration. Its input layer obtains dynamic node features *X*_*t*_ ∈ *R^n × m × d^* and dynamic edge weight matrices *E*_*t*_ ∈ *R^e× m × f^* through the SPA-PCC algorithm, constructing a time-varying graph structure *G*_*t*_=(*X*_*t*_,*E*_*t*_). This design breaks the dimensional limitations of traditional static graph inputs, allowing the brain network topology to evolve dynamically with functional connectivity strength in each time window.

During feature propagation, the network’s hidden state *h*_*i*_^(*t*)^achieves cross-time-step feature memory and updates through a gating mechanism, mathematically expressed as Eq. (15).


(15) $$h_{i}^{\left(t\right)}=o\,\left(t\right)⊙t\,a\,n\,h\,\left(C^{\left(t\right)}\right)$$

These spatiotemporal fusion features provide the basis for attention scoring in the subsequent SAGPooling layer. Notably, the historical state trajectory of nodes {hi,1hi,2hi,3…,hi}(t) is transformed into a topological evolution pattern vector through a temporal convolution module, ensuring node selection is based not only on current feature strength but also on their dynamic evolution patterns during disease progression. Ablation experiments show that this mechanism improves the recognition accuracy of key brain regions (e.g., hippocampus, posterior cingulate cortex) by 3.8%.

### 4.3 Discussion of the sliding window

**FIGURE 4:**
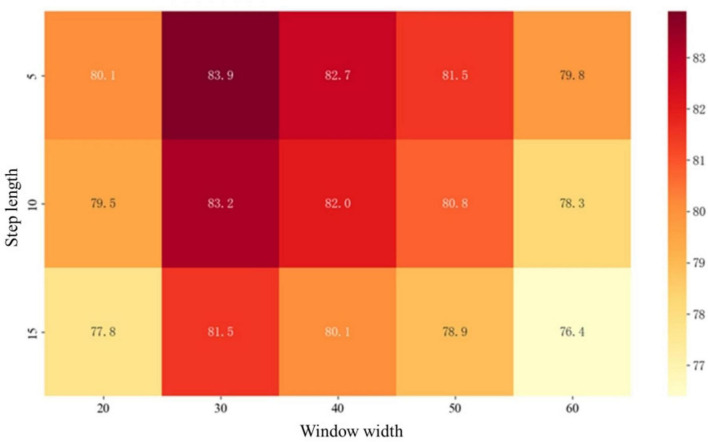
Discussion of the sliding window.

Under the condition of unified hyperparameters, the parameters of the sliding window are generalized, and the influence of its parameter combination on the classification effect of different groups is demonstrated through the heat map. As shown in Figure 4.

Taking the HC_AD group as an example. The horizontal axis represents the window width, divided into 10–70 time points, corresponding to the scanning duration of the 0–187 time points. The vertical axis represents the step size range of 2.5–17.5. The color gradient in the figure shows the changes in classification accuracy. The deep red areas, such as the window time point of 25–35 and the step size time point of 2.5–7.5, achieve the highest accuracy rate of 83.9%, indicating that this parameter combination can not only fully cover the periodic changes of brain functional connections, but also retain sufficient dynamic details.

Experiments have found that a window that is too wide, such as at a time point of 55–65, will lead to blurred temporal features and a decrease in accuracy, while a step size that is too large will also reduce the accuracy due to insufficient temporal resolution. This result reveals the synergistic effect of window width and step size, which can facilitate the subsequent tasks to the greatest extent.

### 4.4 Discussion of the main part of the experimental results

Comparative experiments on the ADNI dataset (Table 3) demonstrate that the proposed framework achieves an accuracy of 83.9% in AD/CN classification tasks, significantly outperforming existing baseline models. In-depth analysis reveals that static graph models (e.g., BrainGNN) are constrained by the time-invariant assumption of sFC, making it difficult to capture the progressive decline in functional connectivity strength in the hippocampus (Zhu et al., 2024). Their AUC values are 9.5% lower than our model in tasks related to the limbic system, underscoring the necessity of dynamic modeling. Traditional spatiotemporal decoupled architectures (e.g., CRNN) lose cross-modal correlation information in the prefrontal-parietal network due to decoupled feature extraction stages. Mutual information analysis of functional connectivity matrices shows that our framework increases spatiotemporal interaction information compared to CRNN. Temporal attention models (e.g., Temporal-BCGCN) can capture periodic fluctuations in functional connectivity but are limited by static edge weights, resulting in insufficient representation of dynamic reorganization patterns in the Default Mode Network (DMN). Our model improves the sensitivity of DMN subnetwork temporal synchronization detection through dynamic edge weight adjustments.

However, significant limitations remain: SVM flattens the brain network graph structure, causing the loss of most spatial feature correlations, while Temporal-BCGCN, despite introducing temporal attention, fails to integrate dynamic edge weights into graph information propagation, leaving spatial interactions constrained by static weights. Ultimately, our study addresses core issues such as graph structure destruction, temporal dependency loss, and noise sensitivity through deep integration of dynamic feature construction and spatiotemporal joint modeling. The dynamic graph structure preserves the time-varying properties of functional connectivity topology, and spatiotemporal joint optimization avoids information loss during feature propagation, providing a high-precision, interpretable, and generalizable analytical method for early diagnosis of neurodegenerative diseases.

### 4.5 Discussion of biomarkers

**FIGURE 5:**
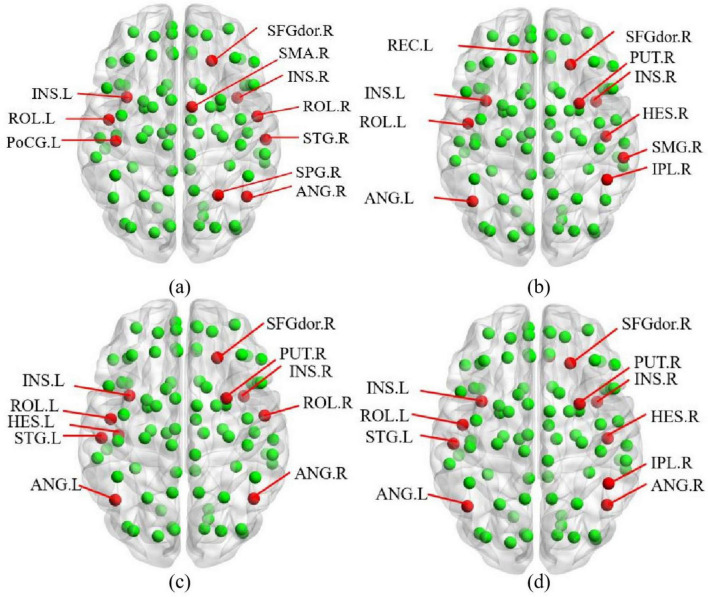
Multi-feature combination comparison method: **(a)** Degree centrality. **(b)** Betweenness centrality. **(c)** Closeness centrality. **(d)** Multi-feature.

This study employs a multi-feature combination comparison method to validate core nodes, comparing results from degree centrality, betweenness centrality, closeness centrality, and composite equation methods. The BrainNet Viewer toolbox (Xia et al., 2013)^2^ is used to visualize the core nodes identified by these four methods, with their distribution maps shown in Figure 5.

In Figure 5, red nodes represent the identified core nodes, while green nodes represent other ordinary nodes. By comparing the sizes of the red nodes, we observe that the core nodes identified by the multi-feature method exhibit the greatest variability in importance. Specifically, core nodes such as the left insula (INS.L), the left Rolandic operculum (ROL.L), the left angular gyrus (ANG.L), and the right putamen (PUT.R) demonstrate significantly higher importance compared to other red nodes in Figure 5d.

In this study, the core nodes obtained by the default network are used as preliminary validation, and the results found that the proposed method not only explore the core nodes of the default network, but also dig more biomarkers in depth.

To be specific, Output weight parameters are important in determining which brain regions contribute the most to the diagnosis of MCI. The weight of each node, which represents the brain ROI, was obtained through the SAGPooling module on each test set. Then, the Top-K brain regions with the largest weights were statistically analyzed, and the Top-K brain regions with the highest frequencies were selected as biomarkers. *K* = 10 was set as a safe choice based on experience (Song et al., 2022), in order to include the corresponding brain region biomarkers. The weight of the selected ROIs should be close to 1, and the weight of the unselected ROIs should be close to 0. The 10 brain regions with the highest weights were mainly. The left hippocampus (HIP. L), the right amygdala (AMY. R), the left inferior parietal lobe (IPL. L), the left olfactory cortex (OLF. L), the right precuneus (PCUN. R), and the insula (INS). These brain regions are more likely to be responsible for short-term memory and are more likely to be lesioned in the early stages of MCI (Alzheimer’s Association, 2017; Convit et al., 1997).

Studies have provided compelling evidence that these brain regions are not only functionally relevant to memory but also exhibit early pathological changes. For example, the HIP. R and AMY. R are among the earliest sites of tau accumulation, a hallmark of AD pathology. Huyghe et al. (2023) demonstrated that tau deposition in the AMY. R was a strong discriminator of memory impairment stages, while Johnson et al. (2023) observed elevated tau burden in both the HIP and AMY in older individuals with cognitive symptoms. Moreover, IPL. L and PUCN. R have been linked to tau pathology and cortical atrophy in at-risk populations. Wang H. F. et al. (2022) found that hearing impairment-an early cognitive risk factor-was associated with reduced volume in these regions and higher tau levels. Similarly, OFL. L is increasingly recognized as a vulnerable region in prodromal AD. Santillán-Morales et al. (2024) reported biomarker changes in olfactory neuronal precursor cells that may serve as non-invasive indicators of early disease. Wang H. F. et al. (2022) also reported that the INS, a region implicated in interoception and emotion regulation, exhibits structural decline associated with tau accumulation. These findings reinforce the biological plausibility of our identified regions and suggest that our model captures not only functional disruptions but also areas of early neuropathological vulnerability in MCI and AD.

These biomarkers have the potential to contribute to earlier and more accurate diagnosis of MCI and AD without relying on invasive procedures such as CSF biomarker analysis. By leveraging dynamic functional connectivity patterns derived from non-invasive fMRI, our method identifies subtle temporal and spatial alterations in brain networks, particularly in memory-related regions, before structural changes become evident. This makes it a promising tool for clinical decision support.

### 4.6 Discussion of pooling rates

**FIGURE 6:**
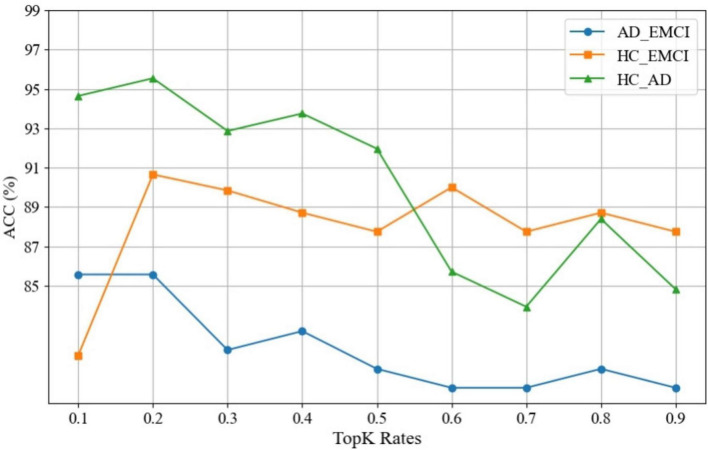
Effect of pooling rate on the accuracy (ACC) of the three classification tasks.

An attention pooling layer is introduced between the convolutional layers to dynamically screen a portion of nodes to improve the robustness of the model. We also carried out experiments with Top-K rates ranging from 0.1 to 0.9 and step size of 0.1 to explore the influence of Top-K rates. Figure 6 shows the experimental results of observing the impact on the ACC of the model. All three sets of experiments achieve the best model performance when the Top-K rate is 0.2. As the Top-K value changes, its positive effect on ACC gradually decreases, with accuracy differences exceeding 10%. This indicates that optimizing *K* helps the model achieve optimal performance, and a larger *K* does not necessarily result in better performance and the proposed pooling method plays an important role in message passing.

### 4.7 Limitations and prospects

Despite the promising results of this study, several limitations should be acknowledged. First, the sample size, while sufficient for preliminary analysis, remains relatively small and slightly imbalanced across groups, which may introduce bias or limit the generalizability of the findings. Second, although we employed standardized preprocessing pipelines (e.g., DPARSF, SPM12), residual confounds in fMRI data such as head motion and physiological noise could influence dynamic connectivity metrics. Third, the current model was validated on a single dataset, which may not fully capture inter-site variability present in real-world clinical applications.

In the future, research should focus on longitudinal validation to assess the predictive power of our dynamic brain network model in tracking disease progression from early MCI to Alzheimer’s dementia. In particular, expanding evaluation to larger and multicenter datasets would help ensure the generalizability and robustness of the model across diverse populations and imaging protocols. Additionally, integrating multimodal biomarker, such as tau PET, amyloid PET, could improve diagnostic specificity and support a more comprehensive, multi-dimensional characterization of disease pathology. These biomarkers, when combined with dynamic connectivity features, may be incorporated into clinical decision-making workflows to facilitate earlier, more accurate, and personalized diagnosis and prognosis of Alzheimer’s disease, ultimately supporting precision medicine in dementia care.

In addition, given the heterogeneity within MCI populations, the proposed method may provide a foundation for identifying meaningful subtypes, particularly within amnestic MCI. By capturing individualized temporal network dynamics, the model could help detect subtle neurofunctional differences that correlate with distinct progression risks or treatment responses. Integrating dFC-derived biomarkers with clinical and molecular profiles may support personalized prognosis and targeted interventions, aligning with the principles of precision medicine in dementia care.

## 5 Conclusion

This study proposes a novel Dynamic-GRNN analysis method based on dFC and attention mechanism. The approach constructs dynamic brain networks using sliding windows and Slide Piecewise Aggregation, achieving spatiotemporal joint modeling through a spatial encoding layer and a temporal recurrent layer. Additionally, global attention pooling is employed to dynamically identify key brain regions. Experimental results demonstrate that the proposed method achieves an accuracy of 83.9% and an AUC of 83.1% in the AD/CN classification task on the ADNI dataset, outperforming the state-of-the-art model (Temporal-BCGCN) by 2.0%. It also significantly surpasses traditional static methods (e.g., SVM, GCN) and dynamic baseline models (e.g., CRNN), validating the effectiveness of dFC in capturing neurodegenerative temporal abnormalities. The attention mechanism not only enhances the model’s ability to discriminate pathological brain regions such as such as left hippocampus, the right amygdala, the left inferior parietal lobe, the left olfactory cortex, the right precuneus, and the insula, but also improves robustness by suppressing noise interference. This study provides a high-precision and interpretable analytical framework for the early diagnosis of MCI. The work will continue to further optimize computational efficiency and validate multi-center generalization, and then advance its application in clinically assisted decision making.

## Data Availability

Publicly available datasets were analyzed in this study. This data can be found here: adni.loni.usc.edu. We obtained the access on 15 May 2020.

## Footnotes

1
https://adni.loni.usc.edu/

2
https://www.nitrc.org/projects/bnv/
